# Quantitative evaluation of a deep learning-based framework to generate whole-body attenuation maps using LSO background radiation in long axial FOV PET scanners

**DOI:** 10.1007/s00259-022-05909-3

**Published:** 2022-07-19

**Authors:** Hasan Sari, Mohammadreza Teimoorisichani, Clemens Mingels, Ian Alberts, Vladimir Panin, Deepak Bharkhada, Song Xue, George Prenosil, Kuangyu Shi, Maurizio Conti, Axel Rominger

**Affiliations:** 1 Advanced Clinical Imaging Technology, Siemens Healthcare AG, Lausanne, Switzerland; 2 Department of Nuclear Medicine, Inselspital, Bern University Hospital, Bern, Switzerland; 3 Siemens Medical Solutions, USA Inc., Knoxville, TN USA

**Keywords:** LAFOV PET, CT-less PET, Deep learning, Attenuation correction, Simultaneous reconstruction

## Abstract

**Purpose:**

Attenuation correction is a critically important step in data correction in positron emission tomography (PET) image formation. The current standard method involves conversion of Hounsfield units from a computed tomography (CT) image to construct attenuation maps (µ-maps) at 511 keV. In this work, the increased sensitivity of long axial field-of-view (LAFOV) PET scanners was exploited to develop and evaluate a deep learning (DL) and joint reconstruction-based method to generate µ-maps utilizing background radiation from lutetium-based (LSO) scintillators.

**Methods:**

Data from 18 subjects were used to train convolutional neural networks to enhance initial µ-maps generated using joint activity and attenuation reconstruction algorithm (MLACF) with transmission data from LSO background radiation acquired before and after the administration of ^18^F-fluorodeoxyglucose (^18^F-FDG) (µ-map_MLACF-PRE_ and µ-map_MLACF-POST_ respectively). The deep learning-enhanced µ-maps (µ-map_DL-MLACF-PRE_ and µ-map_DL-MLACF-POST_) were compared against MLACF-derived and CT-based maps (µ-map_CT_). The performance of the method was also evaluated by assessing PET images reconstructed using each µ-map and computing volume-of-interest based standard uptake value measurements and percentage relative mean error (rME) and relative mean absolute error (rMAE) relative to CT-based method.

**Results:**

No statistically significant difference was observed in rME values for µ-map_DL-MLACF-PRE_ and µ-map_DL-MLACF-POST_ both in fat-based and water-based soft tissue as well as bones, suggesting that presence of the radiopharmaceutical activity in the body had negligible effects on the resulting µ-maps. The rMAE values µ-map_DL-MLACF-POST_ were reduced by a factor of 3.3 in average compared to the rMAE of µ-map_MLACF-POST_. Similarly, the average rMAE values of PET images reconstructed using µ-map_DL-MLACF-POST_ (PET_DL-MLACF-POST_) were 2.6 times smaller than the average rMAE values of PET images reconstructed using µ-map_MLACF-POST_. The mean absolute errors in SUV values of PET_DL-MLACF-POST_ compared to PET_CT_ were less than 5% in healthy organs, less than 7% in brain grey matter and 4.3% for all tumours combined.

**Conclusion:**

We describe a deep learning-based method to accurately generate µ-maps from PET emission data and LSO background radiation, enabling CT-free attenuation and scatter correction in LAFOV PET scanners.

**Supplementary Information:**

The online version contains supplementary material available at 10.1007/s00259-022-05909-3.

## Introduction

Attenuation correction of PET emission data is one of the essential corrections in PET image formation for accurate quantification. In the early generation of PET scanners, attenuation of 511 keV annihilation photons was measured from a separate transmission scan using an external radionuclide-based source (i.e. germanium-68) [1] and an attenuation map (µ-map) was generated. Although this method was able to directly measure the attenuation factors at the same energy with the annihilated photons, it suffered from noisy data and long acquisition times [2]. With the introduction of combined PET/CT systems [3], linear attenuation coefficients (LACs) at 511 keV are estimated from CT images (µ-map_CT_) using a bilinear relationship with Hounsfield unit values [4, 5].

Recently introduced long axial field-of-view (LAFOV) PET/CT systems have enabled total-body PET imaging using a single bed position [6, 7]. In addition to large anatomical coverage that includes major body organs without the need for any bed movement, these systems markedly increase system sensitivity and noise equivalent count rates compared to standard axial FOV (SAFOV) PET scanners [8–11]. Furthermore, LAFOV was shown to provide PET images with superior image quality compared to SAFOV systems. These technological advancements can be utilized in a clinical setting by reducing the activity of the injected radiotracer without compromising the image quality and quantification accuracy [12, 13] and reducing the PET examination time [14–16]. However, the benefits of low-dose PET examinations using LAFOV PET systems can be hindered by the dose associated with the CT scans performed for attenuation correction. While the CT provides important additional diagnostic information and accurate anatomical localization of PET findings, there are potentially numerous situations in which the requirement for CT can be waived: for example, where an anatomical CT scan is available from previous examinations performed during the work-up of the patient. Furthermore, CT-less protocols could be desirable in low dose PET/CT examinations for screening or in paediatric scans to minimize the ionization radiation-induced risks in the health of young patients or in research protocols.

The development of lutetium-based scintillators, such as lutetium oxyorthosilicate (LSO) scintillators [17], and introduction of silicon-based photomultipliers (SiPM) [18] resulted in substantial improvements in coincidence timing resolution with values close to 200 ps [10, 19], increasing the accuracy and robustness of PET image reconstruction process with time-of-flight (TOF) PET reconstruction algorithms [20, 21]. These advances also pushed the potential of methodologies which seek to jointly estimate the activity and attenuation from TOF-PET data [22–24] such as maximum likelihood estimation of attenuation and activity (MLAA) or maximum likelihood estimation of activity and attenuation correction coefficients (MLACF). Previous work has shown that incorporation of prior information, such as anatomical information derived from magnetic resonance imaging (MRI) data or other sources, can be used to improve the robustness of joint reconstruction methods, scatter correction in particular, by providing initial conditions [25–27]. Hwang et al. have shown that MLAA derived µ-maps from a PET/MRI scanner can be used as an input data to a deep learning-based method to synthesize more accurate attenuation maps [28].

The radioisotope lutetium-176 (^176^Lu) found in LSO scintillators of PET detectors decays with a half-life of 38 billion years, emitting gamma rays with 307, 202, and 88 keV during the process [29]. We have previously demonstrated that this LSO background radiation can be detected using a high sensitivity LAFOV PET scanner and developed a method to generate µ-maps using MLACF algorithm with LSO transmission (LSO-TX) data (µ-map_MLACF_) [30]. In this paper, we extend the previous method by incorporating a deep learning-based model to synthesize enhanced whole-body µ-maps (µ-map_DL-MLACF_) based on µ-map_MLACF_ images. We perform a quantitative comparison of µ-maps generated using the proposed deep learning-enhanced MLACF method against µ-maps generated using the MLACF and CT-based methods. Secondly, we evaluate the performance of the proposed method using pre- and post-injection LSO-TX measurements. Finally, we compare the PET images reconstructed using µ-maps based on MLACF-, DL-MLACF-, and CT-based methods and assess their quantitative performance on healthy and malignant tissues.

## Materials and methods

### Patient population

Within this study, 18 oncological patients (age: 60.6 ± 14.7, 12 males/6 females, weight: 76.7 ± 18.5 kg [range: 53–130 kg], body mass index (BMI): 24.9 ± 5.6 kg/m^2^ [range: 17.3–42.0 kg/m^2^]) underwent PET scans as part of standard care PET/CT examinations at the University Clinic for Nuclear Medicine, Inselspital, Bern. The patient demographics together with their diagnoses are included in Table 1. All patients provided written informed consent and the local Institutional Review Board approved the study (KEK 2019–02,193).

**Table 1:** Overview of patient demographics (Ca, cancers)

Patient no	Age (y)	Sex (M/F)	BMI (kg/m^2^)	Weight (kg)	Injected dose (MBq)	Diagnosis
1	44	F	20	55	182	Breast Ca
2	70	F	21	53	167	Lung Ca
3	62	M	24.2	70	213	Lymphoma
4	85	M	24.6	78	249	Lymphoma
5	80	F	32.5	94	275	Fallopian tube Ca
6	77	M	26.1	81	261	Lymphoma
7	29	M	42	130	398	Lymphoma
8	64	M	22.6	80	247	Gastric Ca
9	53	M	22.2	68	208	Lung Ca
10	48	F	18.4	52	171	Breast Ca
11	59	M	26.2	85	276	Lymphoma
12	40	F	17.3	63	191	Cervical Ca
13	54	M	24.9	79	247	Lymphoma
14	66	M	28.4	94	279	Lung Ca
15	51	M	24.2	80	240	Lymphoma
16	68	M	25.5	79	227	Lymphoma
17	73	F	22.5	59	197	Breast Ca
18	68	M	25.5	80	227	Melanoma
Mean ± SD	60.6 ± 14.7		24.9 ± 5.6	60.6 ± 14.7	236.4 ± 53.8	

### Imaging protocol

**Fig. 1:**
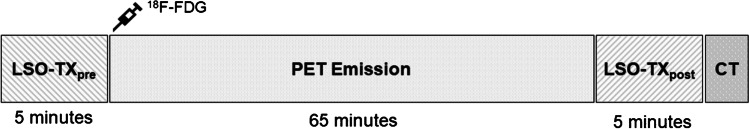
PET protocol used in this study. LSO transmission data were acquired for 5 min just prior to administration of ^18^F-FDG and 65 min post injection

The data used within this work were acquired using a dynamic PET protocol where the tracer administration was performed in the scanner. The protocol is illustrated in Fig. 1. Before the administration of ^18^F-fluorodeoxyglucose (^18^F-FDG) activity, a 5-min long LSO-TX acquisition was performed using a special acquisition protocol with open energy (160–725 keV) and coincidence timing windows (6.64 ns). Following, the ^18^F-FDG was injected from the left or right arm (average activity: 234.6 ± 54.9 MBq, target dose: 3 MBq/kg) and list-mode PET data were acquired for 65 min using Biograph Vision Quadra (Siemens Healthineers, Hoffman Estates, IL, USA) LAFOV PET/CT system. In this work, we only used the PET emission data from 55 to 65 min post injection. After the PET acquisition, a second set of LSO-TX list-mode data was acquired for 5 min (65 to 70 min post injection). At the end of the study, low-dose CT (pitch factor: 1, maximum voltage: 120 kV, maximum tube current 90 mAs, CareDose4D, CarekV) data were acquired as part of the clinical examination. The CT images were reconstructed with a voxel size of 1.52 × 1.52 × 1.65 mm^3^.

### Attenuation maps

**Fig. 2:**
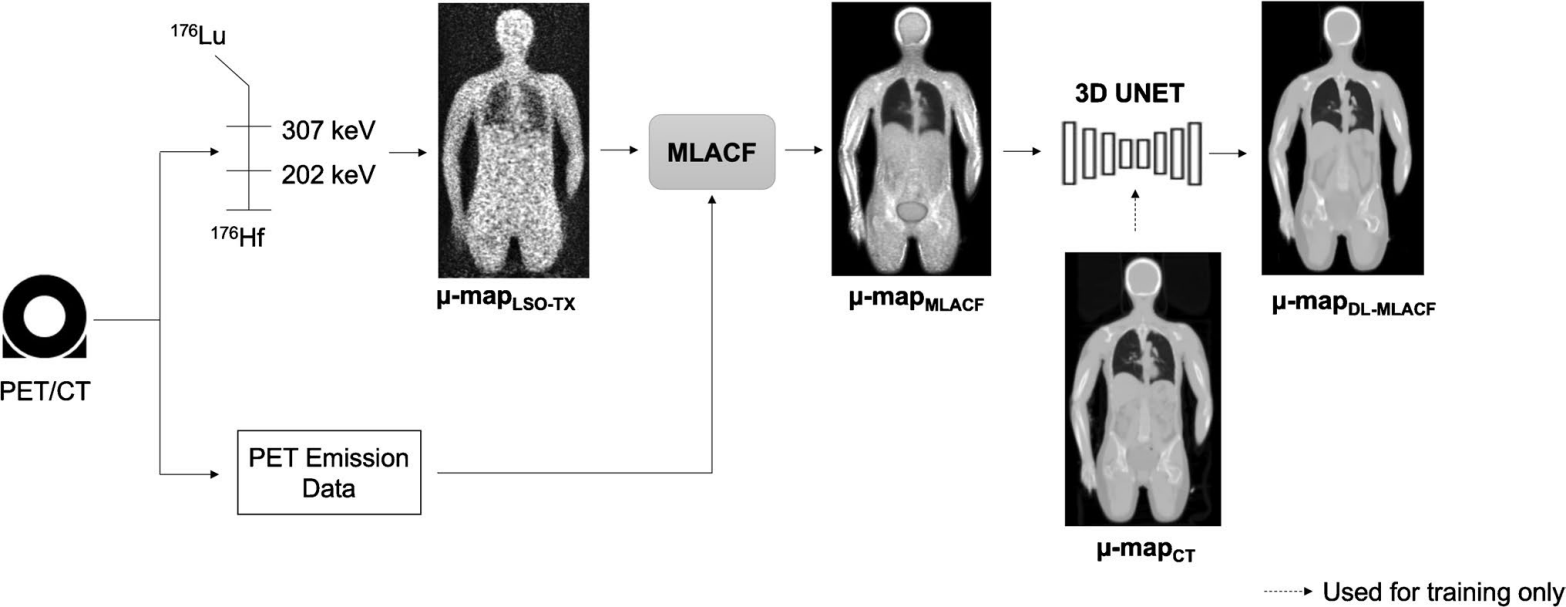
Brief overview of methodology used to generate MLACF- and deep learning-enhanced MLACF-based attenuation maps

Figure 2 depicts the different methods used to generate attenuation maps in this work. CT-based µ-maps were generated by converting the Hounsfield units (HU) of the reconstructed CT images to attenuation correction factors using a bi-linear transformation [5]. These µ-maps were resampled to 440 × 440 × 645 matrix with a voxel size of 1.65 × 1.65 × 1.65 mm^3^, as used in standard PET reconstructions.

#### MLACF-derived µ-maps

The list-mode data acquired with wide-open energy and coincidence-timing windows were post processed and two LSO-TX sinograms, corresponding to LSO-TX at 307 keV and 202 keV, were generated by extracting events using energy windows of 275 to 355 keV and 165 to 247 keV respectively. Two initial µ-maps were reconstructed from these sinograms using maximum likelihood for transmission tomography method [31] with 8 iterations and 3 subsets. These µ-maps were mapped to 511 keV, and then averaged and smoothed using a Gaussian filter with a full width half maximum (FWHM) of 4 mm. The resulting LSO-TX derived µ-map and the TOF emission sinogram were used as inputs to MLACF algorithm to jointly reconstruct a PET image and an MLACF-derived attenuation map using 20 global iterations [30]. Two sets of MLACF-derived attenuation maps were generated by using the LSO-TX data acquired pre- and post- ^18^F-FDG injection, referred as µ-map_MLACF-PRE_ and µ-map_MLACF-POST_ in the rest of the paper respectively. To minimize the effects of motion artefacts, MLACF-derived µ-maps were co-registered to CT-derived µ-maps by applying a combination of rigid and non-rigid registration using NiftyReg package [32]. The bending energy weight was set to 0.1% to constrain the degrees of freedom of the non-rigid deformation [33].

#### Deep learning based µ-maps

Convolutional neural networks (CNNs) were trained to enhance the MLACF-derived µ-maps using the paired µ-map_CT_ as target images. To achieve this, we used a three dimensional UNET architecture [34, 35] with five down-sampling and five up-sampling layers, and parametric rectified linear unit (PReLU) used as the activation function. Multiple patches with a matrix size of 64 × 64 × 64 were used in the training. The input images were normalized to zero mean and unity variance. We performed data augmentation by randomly applying ± 20% image scaling and ± 10% image rotation. We trained and tested the networks using fivefold cross-validation, where for each fold, the data were split to 14 training (78% of the data) and 4 testing sets (22% of the data). Separate models were trained using µ-map_MLACF-PRE_ and µ-map_MLACF-POST_ images as input images and same cross-validation folds were used across these models. The predicted attenuation maps from models trained using µ-map_MLACF-PRE_ and µ-map_MLACF-POST_ images are referred as µ-map_DL-MLACF-PRE_ and µ-map_DL-MLACF-POST_ respectively.

### PET image reconstruction

The PET emission data from 55 to 65 min post injection were reconstructed using µ-map_MLACF-PRE_, µ-map_MLACF-POST_, µ-map_DL-MLACF-PRE_, µ-map_DL-MLACF-POST_ and µ-map_CT_ for each subject. The PET images reconstructed using the different µ-map methods are referred as PET_MLACF-PRE_, PET_MLACF-POST_, PET_DL-MLACF-PRE_, PET_DL-MLACF-POST_, and PET_CT_. The PET images were reconstructed with PSF + TOF algorithm using 4 iterations and 5 subsets using a dedicated image reconstruction software prototype (e7-tools, Siemens Healthineers). The emission data were corrected for decay, randoms, and scatter. The image matrix was set to 440 × 440 × 645 with a voxel size of 1.65 × 1.65 × 1.65 mm^3^. A Gaussian post-reconstruction filter was applied with a FWHM of 2 mm.

### Data analysis

The generated attenuation maps and PET images were evaluated using regional analyses. The percentage relative mean error (rME) and relative mean absolute error (rMAE) values were calculated using Eqs. 1 and 2:

1 $$rME\left(\%\right)=100 \frac{{I}_{x}-{I}_{ref}}{{I}_{ref}}$$

2 $$rMAE\left(\%\right)=100 \frac{\left|{I}_{x}-{I}_{ref}\right|}{{I}_{ref}}$$

where I_x_ represents µ-maps generated using MLACF- or deep learning-based methods or PET images reconstructed using these µ-maps. Similarly, I_ref_ represents µ-map_CT_ or PET_CT_.

The µ-map_CT_ images were segmented into 3 VOIs: water-based soft tissue, fat-based soft tissue and bones using a thresholding algorithm. Bones were segmented by only including voxels with a LAC greater than 0.105 cm^−1^ followed by a flood-fill operation to include the bone marrow in the segmentations. Fat- and water-based soft tissue segmentations were obtained by thresholding voxels with LAC values outside 0.080–0.090 cm^−1^ range and 0.090–0.105 cm^−1^ range respectively. Furthermore, three dimensional segmentations of liver, lungs, kidneys, spleen, grey and white matter of the brain were obtained using a semi-automatic method [36, 37]. Hypermetabolic tumour lesions (*n* = 24) were delineated by a qualified nuclear medicine physician using an isocontour tool (PMOD 4.1, threshold set to 50% of max value).

### Statistical tests

Nonparametric two-sided Wilcoxon signed-rank tests were used to assess differences between different µ-maps and reconstructed PET images. Statistically significance was considered for *P*-values lower than 0.05. Spearman’s rank correlation was used to assess any potential relationship between the accuracy of the method and patient BMI and Spearman’s rank coefficient (r_s_) and *P*-values are reported.

## Results

**Fig. 3:**
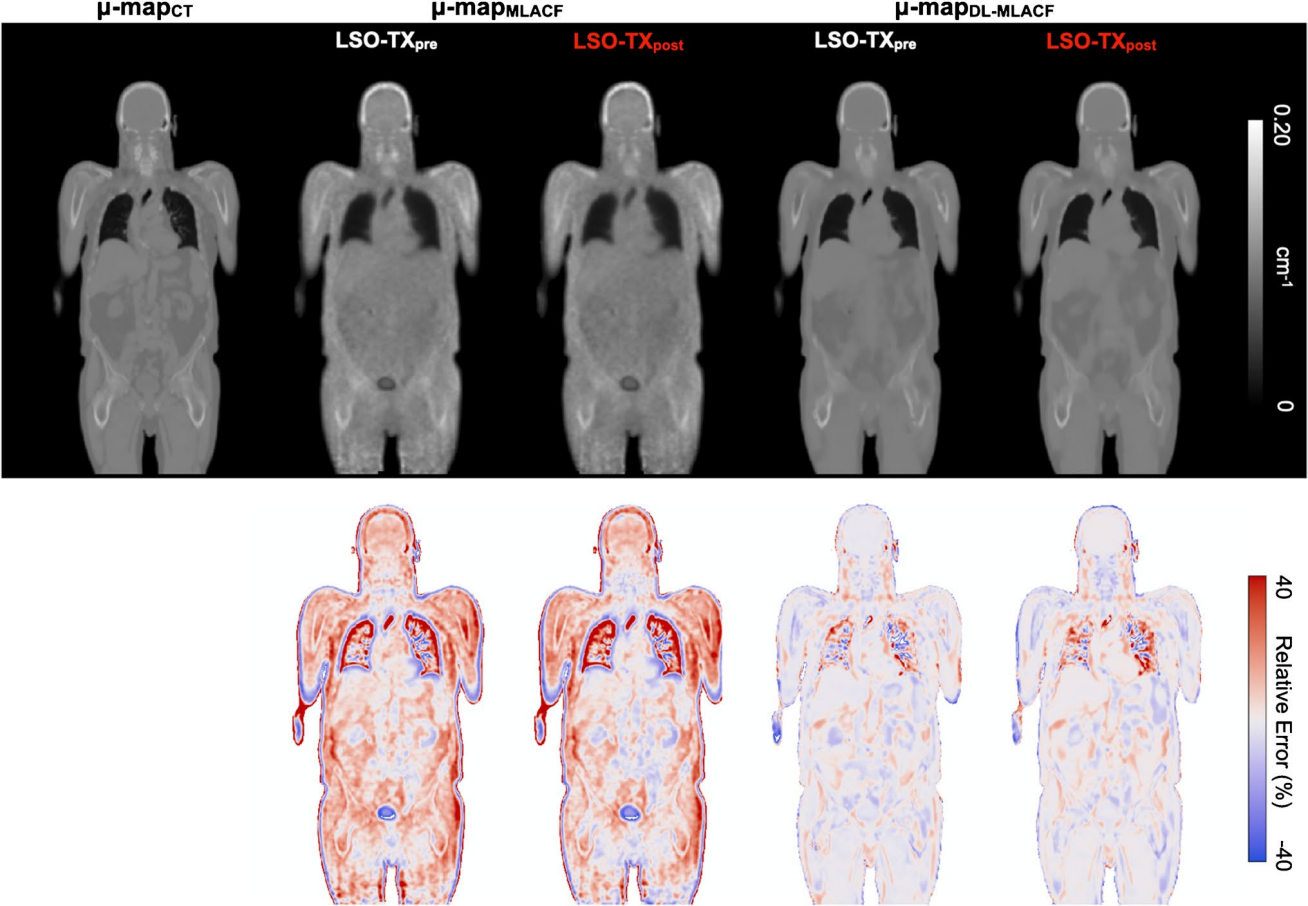
Top row: attenuation maps of a representative subject generated using the CT-, MLACF-, and deep learning-enhanced MLACF-based methods. Attenuation maps from pre- and post-injection LSO-TX acquisitions are shown separately. Bottom row: voxelwise maps of relative error  distribution of MLACF- and DL-MLACF-based µ-maps relative to CT-based µ-map

Attenuation maps generated using CT- (µ-map_CT_), MLACF- (µ-map_MLACF-PRE_ and µ-map_MLACF-POST_) and deep learning-enhanced MLACF (µ-map_DL-MLACF-PRE_ and µ-map_DL-MLACF-POST_) methods and corresponding rME maps for a representative subject are shown in Fig. 3. There were no visual differences between µ-map_MLACF-PRE_ and µ-map_MLACF-POST_, and between µ-map_DL-MLACF-PRE_ and µ-map_DL-MLACF-POST_. The µ-maps generated using the MLACF-based method had some artefacts, where the attenuation correction factors in the skull, skin, and bladder of the patient were overestimated. These artefacts were significantly improved in the µ-maps generated using the deep learning-based method. In overall, the deep learning-enhanced MLACF method produced µ-maps with less noise and a good visual resemblance to µ-map_CT_.

**Fig. 4:**
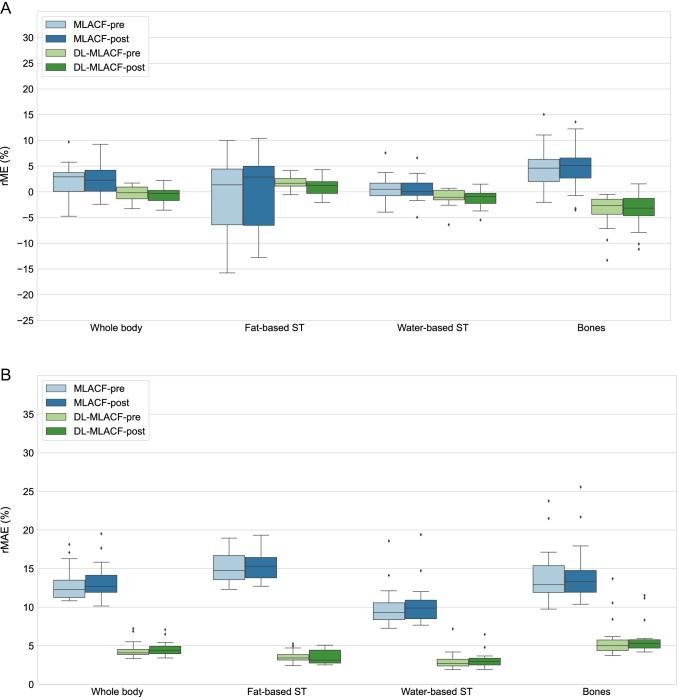
Box-and-whisker plots of VOI-based %rME and %rMAE for different attenuation maps. For each box, edges represent 25th and 75th percentiles and whiskers represent rest of the distribution without the outliers. Central horizontal line mark represents the median. Outliers are plotted using individual points

These findings were further validated with the quantitative VOI-based assessments shown in Fig. 4. It is shown that the rMAE was reduced by a factor of 4.3 in fat-based soft tissue, 3.3 in water-based soft tissue and 2.4 in bones in µ-map_DL-MLACF-POST_ compared to µ-map_MLACF-POST_. Similar improvements were also seen between µ-map_DL-MLACF-PRE_ and µ-map_MLACF-PRE_ images. No significant differences were seen between the µ-map_DL-MLAC-PRE_ and µ-map_DL-MLACF-POST_ rME values (*P* = 0.29 in fat-based soft tissues, *P* = 0.16 in water-based soft tissues and *P* = 0.28 in bones), suggesting that the presence of radiopharmaceutical activity did not induce any major artefacts in the resultant attenuation maps. The µ-map_DL-MLACF-POST_ had an rMAE of 3.6% in fat-based soft tissues, 3.2% in water-based soft tissues and 6.0% in bones. The rMAE values for water-based soft tissues and bones were 2-times higher for the patient with a BMI of 42 kg/m^2^, which was an outlier in terms of rME and rMAE values. The µ-maps of this patient and another larger patient with a BMI of 32.5 kg/m^2^ are illustrated in supplementary Figs. 1 and 2 respectively.

**Fig. 5:**
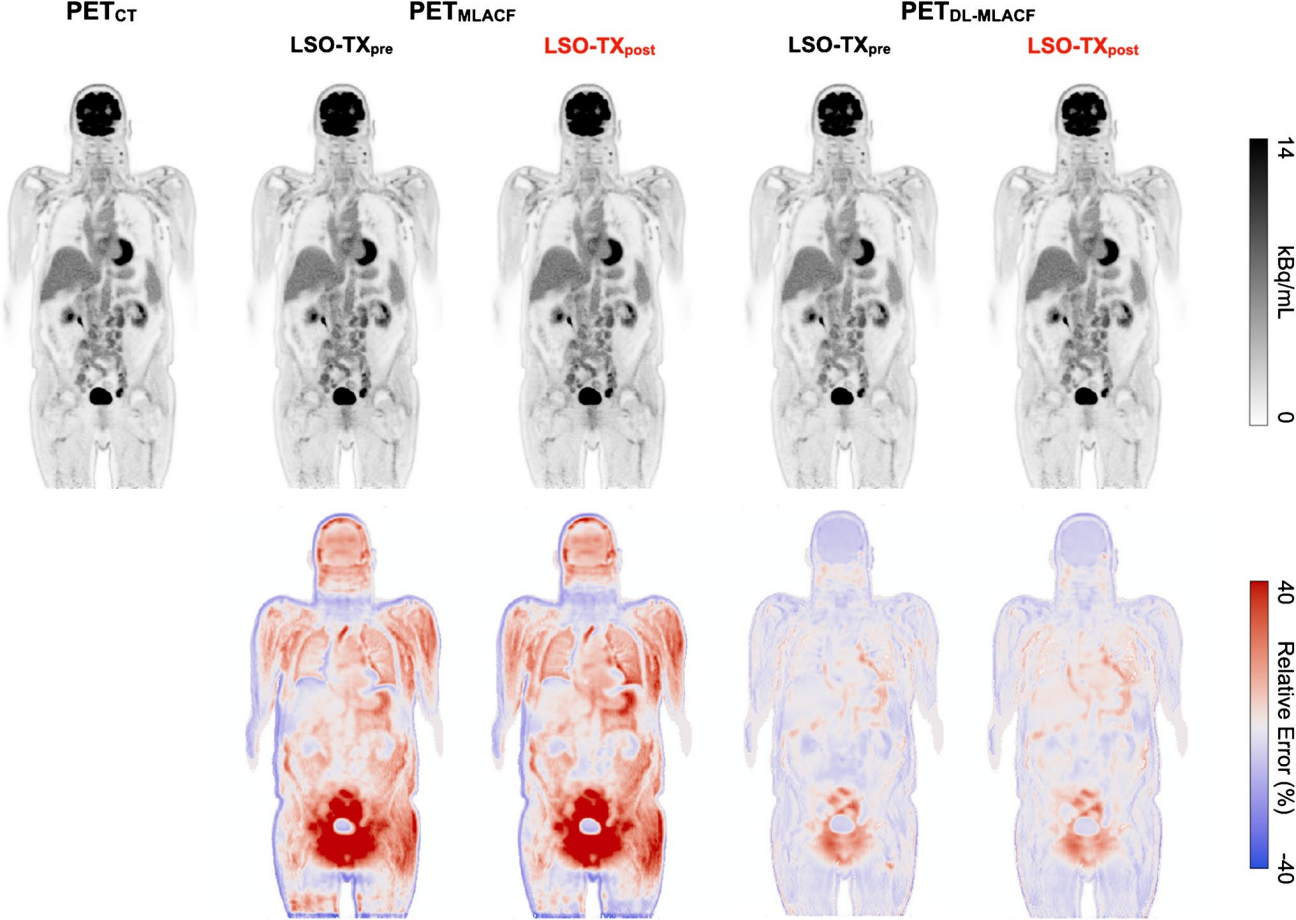
Top row: PET images of a representative subject reconstructed using the CT-, MLACF-, and deep learning-enhanced MLACF-based attenuation maps. PET images reconstructed using MLACF- and DL-based µ-maps generated using pre- and post-injection LSO-TX data are shown separately. Bottom row: voxelwise maps of relative error distribution of PET images relative to the PET image reconstructed using the CT-based µ-map

**Fig. 6:**
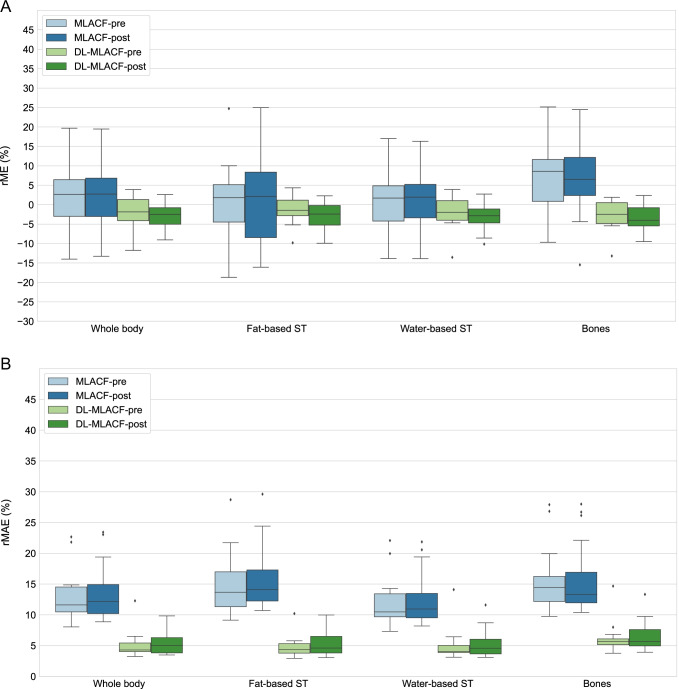
Box-and-whisker plots of VOI-based %rME and %rMAE for reconstructed PET images. For each box, edges represent 25th and 75th percentiles and whiskers represent rest of the distribution without the outliers. Central horizontal line mark represents the median. Outliers are plotted using individual points

Figure 5 shows PET images of a representative subject reconstructed using CT-, MLACF-, and deep learning-enhanced MLACF µ-maps together with their rME maps. The PET_DL-MLACF-PRE_ and PET_DL-MLACF-POST_ images closely resembled the PET_CT_ images. The VOI-based rMAE results showed a 3.0-times reduction in fat-based soft tissue, 2.4-times reduction in water-based soft tissue and 2.5-times reduction in bones in PET_DL-MLACF-POST_ compared to PET_MLACF-POST_ images (Fig. 6). Similar to µ-map results, no significant difference was observed between VOI-based rMAE values of PET_DL-MLACF-POST_ and PET_DL-MLACF-PRE_ images (*P* = 0.78 in fat-based soft tissue, *P* = 0.91 in water-based soft tissue and *P* = 0.98 in bones).

**Fig. 7:**
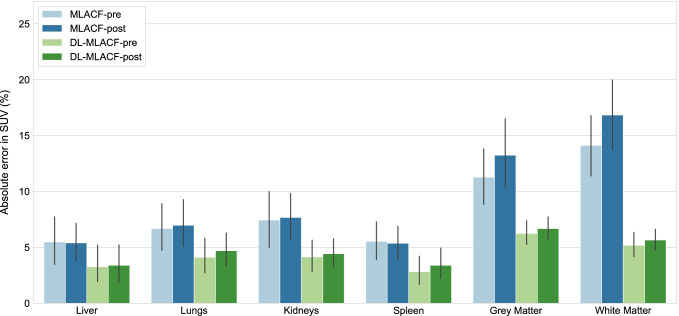
Bar plot of percentage absolute error in SUV_mean_ values of multiple organs of interest and brain grey and white matter from PET images reconstructed using pre- and post- injection MLACF- and deep learning-enhanced MLACF-based µ-maps compared to PET images reconstructed using CT-based µ-maps. Error bars indicate the standard deviation of the dataset

**Fig. 8:**
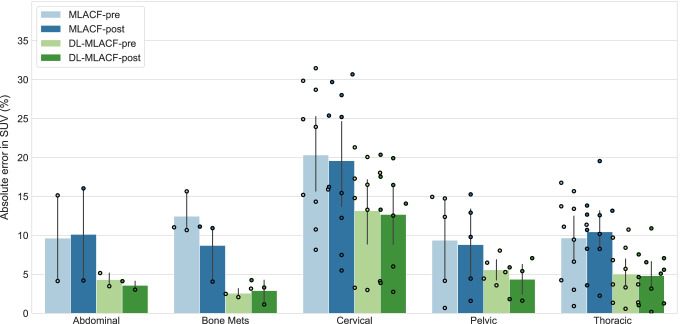
Bar plot of percentage absolute error in SUV_mean_ values of different tumours in PET images reconstructed using pre- and post-injection MLACF- and deep learning-enhanced MLACF-based µ-maps compared to PET images reconstructed using CT-based µ-maps. Error bars indicate the standard deviation of the dataset. Data points are also plotted individually

Figure 7 illustrates the average percentage error in SUV_mean_ values in organs of interest and brain grey and white matter. The PET_DL-MLACF-POST_ achieved an average absolute error of less than 4% in the liver and spleen, 4.7% in the lungs, 6.7% in the grey matter, and 5.6% in the white matter of the brain. Figure 8 shows the absolute errors in SUV_mean_ of tumour lesions, grouped per their anatomical location. Bone lesions showed a 3.2-times absolute error reduction for PET_DL-MLACF-POST_ compared to PET_MLACF-POST_, where thoracic lesions demonstrated a 2.7-times absolute error reduction. In average, the PET_DL-MLACF-POST_ achieved an absolute percentage error of 3.6% in abdominal, 2.9% in bone, 4.4% in pelvic, and 4.8% in thoracic lesions. We observed larger errors in cervical lesions for all methods, where the mean absolute error was 12.7% for PET_DL-MLACF-POST_ and 12.9% for PET_DL-MLACF-PRE_. However, it should be noted that these results were highly influenced by the values from the patient with an outlier BMI of 42 kg/m^2^ with seven cervical lesions. Excluding this subject, the average absolute error was 4.2% for PET_DL-MLACF-POST_ and 3.5% for PET_DL-MLACF-PRE_. The absolute error, averaged across all tumours excluding the outlier patient, was reduced from 9.6 to 4.3% for PET_DL-MLACF-POST_ compared to PET_MLACF-POST_ with a statistically significant difference between methods (*P* < 0.001). All tumours combined, no significant difference was observed between PET_DL-MLACF-PRE_ and PET_DL-MLACF-POST_ (*P* = 0.23).

As also described above, we observed relatively larger errors in µ-maps and reconstructed PET images of the patient with a BMI of 42.0 kg/m^2^ when MLACF and DL-MLACF methods are used (images shown in supplementary Figs. 1 and 2). The results of the correlation analysis between BMI and whole-body %rMAE values in PET images showed a weak positive association for PET_MLACF-PRE_ (*r*_*s*_ = 0.35, *P* = 0.15) and a positive correlation for PET_MLACF-POST_ (*r*_*s*_ = 0.45, *P* < 0.05). For the deep learning-based methods, weak positive associations with BMI values were present for both PET_DL-MLACF-PRE_ (*r*_*s*_ = 0.32, *P* = 0.19) and PET_DL-MLACF-POST_ (*r*_*s*_ = 0.16, *P* = 0.52). Scatter plots of BMI and whole-body %rMAE values of PET images reconstructed with different µ-maps are shown in supplementary Fig. 3. Contrary to 9.8% whole-body %rMAE present in the PET_DL-MLACF-POST_ of the patient with a BMI of 42.0 kg/m^2^, 3.8% whole-body %rMAE was present in the PET_DL-MLACF-POST_ images of the patient with second highest BMI of 32.5 kg/m^2^ (µ-maps and PET images are shown in supplementary Figs. 4 and 5).

## Discussion

The introduction of LAFOV PET scanners with increased system sensitivity compared to SAFOV PET scanners opens opportunities for low-dose PET imaging protocols. Although the risks of the equivalent dose associated with nuclear medicine imaging are modest [38], there remains sufficient concern to warrant a number of studies exploring the potential for lower activity PET scans without compromising image quality via a number of approaches [39, 40]. However, the value of low-dose PET imaging protocols can be hindered by the CT scans required for attenuation correction. Although CT is a critical part of most clinical PET/CT studies and delivers important anatomical and diagnostic information to the interpreting physician, further reductions in the patient dose through omission of the CT component could find utility in some specific clinical scenarios. For instance, a CT-less method for PET attenuation correction might be desirable in longitudinal or follow-up PET scans where a CT scan is already available from the patient’s work up. The higher sensitivity of LAFOV systems can be exploited for acquisition of images at later time points [41], dual-time-point studies [42, 43], or as part of abbreviated dynamic imaging protocols [44]. It can also be used for dose reduction in neuroimaging studies where an MR scan is often available for anatomical information or to reduce radiation exposure in cancer screening and paediatric studies.

In this work, we exploited the high sensitivity of a LAFOV PET system to detect LSO-TX events and used a joint reconstruction and deep learning-based method to construct attenuation maps from the LSO-TX data. Qualitative and quantitative analyses indicate that the deep learning-enhanced MLACF method was able to generate µ-maps with better resemblance to CT-based µ-maps than the µ-map_MLACF_, particularly improving the overestimation of the attenuation coefficients in the skin and skull of the patients, addressing the crosstalk issues around the bladder, and reducing the noise present in µ-map_MLACF_. PET images reconstructed with µ-map_DL-MLACF-PRE_ and µ-map_DL-MLACF-POST_ showed less than − 3.6% rME in fat-based soft tissue, water-based soft tissue, and bones. Furthermore, mean organ and tumour SUV values calculated from PET_DL-MLACF-PRE_ and PET_DL-MLACF-POST_ images had less than 7% absolute error compared to mean SUV values from PET_CT_ images. Quantitative VOI-based comparisons showed no significant differences between µ-map_DL-MLACF-PRE_ and µ-map_DL-MLACF-POST_. These results indicate that the presence of PET activity had negligible effect on the quality of LSO-TX images and the proposed method achieved comparable performance with pre- and post-injection LSO-TX data. The LSO-TX data can also be acquired simultaneously with PET emission data, in our case reducing the total scan duration to five minutes.

The use of deep learning-based methods in PET attenuation correction has been increasingly popular, particularly in PET/MRI imaging where lack of CT-based attenuation maps introduced significant challenges to accurate PET quantification [45]. In previous work, CNNs were trained using co-registered MR and CT images to generate pseudo-CT based µ-maps for head [46, 47] and pelvis [48–50], which were shown to be more accurate compared to vendor-provided atlas based µ-maps. Besides, the use of supervised deep learning techniques such as CNN has limited performance in generating whole-body µ-maps as these techniques require perfectly aligned MR and CT whole-body images which is not straightforward. As an alternative, unsupervised methods with cycle-consistent GAN architecture were used to generate attenuation-corrected PET images from non-attenuation-corrected PET images [51, 52]. Most related to our work, Hwang et al. [28] generated whole-body µ-maps using a CNN and initial µ-maps generated using MLAA joint reconstruction algorithm with TOF emission data. However, the lack of an initial attenuation and emission images can cause challenges in scatter correction during the joint reconstruction process and can lead to unscaled µ-maps with inaccurate attenuation factors [27]. In a more recent work, Hwang et al. proposed incorporating non-attenuation-corrected PET images in their method to estimate the scatter distribution [53]. Here, we suggest use of an LSO-TX derived to µ-map to provide initial conditions for scatter correction in the MLACF joint reconstruction algorithm.

In this work, we used CT-based µ-maps as target images during the training and evaluation of the methodology. While CT-based PET attenuation correction is often considered the gold standard, it can also suffer from some limitations. Truncation or beam-hardening artefacts can be introduced to CT images when the patient’s arms are present in the field-of-view [54]. This is particularly an issue for patients with large BMIs [3]. Previous work has also shown that the use of CT-based AC can lead to some bias in linear attenuation coefficients of cancellous and compact bones, albeit the minor PET quantification errors caused by this might only be clinically significant in quantitative bone studies [55]. Furthermore, potential patient motion and the respiratory movement of the chest between PET and CT acquisitions can lead to spatial mismatch of images which can lead to incorrect PET attenuation correction factors [56]. In this work, to make a fair comparison, MLACF and DL-MLACF based µ-maps were co-registered to their CT pairs. However, it can be argued that the proposed method is less prone to misregistration errors when the LSO-TX data is simultaneously acquired with the PET emission data. Further evaluation with phantom data is required to assess the performance of our method in such scenarios.

Another limitation of this study was the relatively small sample size of our training set. In this work, we used cross-validation to train and test our method using data from 18 subjects. Since one of the aims of this work was to evaluate the accuracy of the proposed method with LSO-TX data acquired pre- and post-tracer administration, the data used in this study were acquired using a dynamic ^18^F-FDG protocol where the tracer administration was performed in the scanner. The logistical challenges of these lengthy dynamic scan protocols limited the size of our study cohort. In principle, the size of the training set can be increased in future studies using only post-injection LSO-TX data. We observed larger errors for one subject whose BMI was 41% above the average population BMI, suggesting that the proposed method might have limited performance in very large patients (i.e. BMI > 40 kg/m^2^). This can be addressed in future work by enlarging the data pool and including more diverse population of patient data (i.e. larger patients) in the model training. Furthermore, the MLACF-based µ-maps which are used as the only input to our model were jointly reconstructed using LSO-TX and ^18^F-FDG emission data. Further investigation is needed to assess the performance of our method with other PET radiotracers. Finally, introduction of LAFOV PET/CT systems demostrates great potential in reducing patient dose in PET examinations. Further work includes evaluation of the method in PET scans with lower injected activities of radiopharmaceuticals.

## Conclusion

We present the development and initial validation of a deep learning-based method to synthesize CT-free attenuation maps using information from LSO transmission and PET emission data. We demonstrated that the proposed method was able to generate accurate attenuation maps, independent of the timing of the LSO-TX scan, with strong correlation to CT-based attenuation maps. Results presented in this work suggest that the proposed method can enable CT-free quantitative PET imaging which might be beneficial in certain clinical scenarios and research studies.

## Supplementary Information

Below is the link to the electronic supplementary material.

Supplementary file1 (DOCX 20.1\4 MB)
