# Supplementary material for: Quantitative evaluation of a deep learning-based framework to generate whole-body attenuation maps using LSO background radiation in long axial FOV PET scanners
Source: Eur J Nucl Med Mol Imaging. 2022 Jul 19;49(13):4490–502. doi: 10.1007/s00259-022-05909-3 (PMC9606046; doi:10.1007/s00259-022-05909-3)
Supplement: Supplementary file 1 — Supplementary file1 (DOCX 20.1\4 MB) [file 259_2022_5909_MOESM1_ESM.docx]

**SUPPLEMENTARY FIGURES
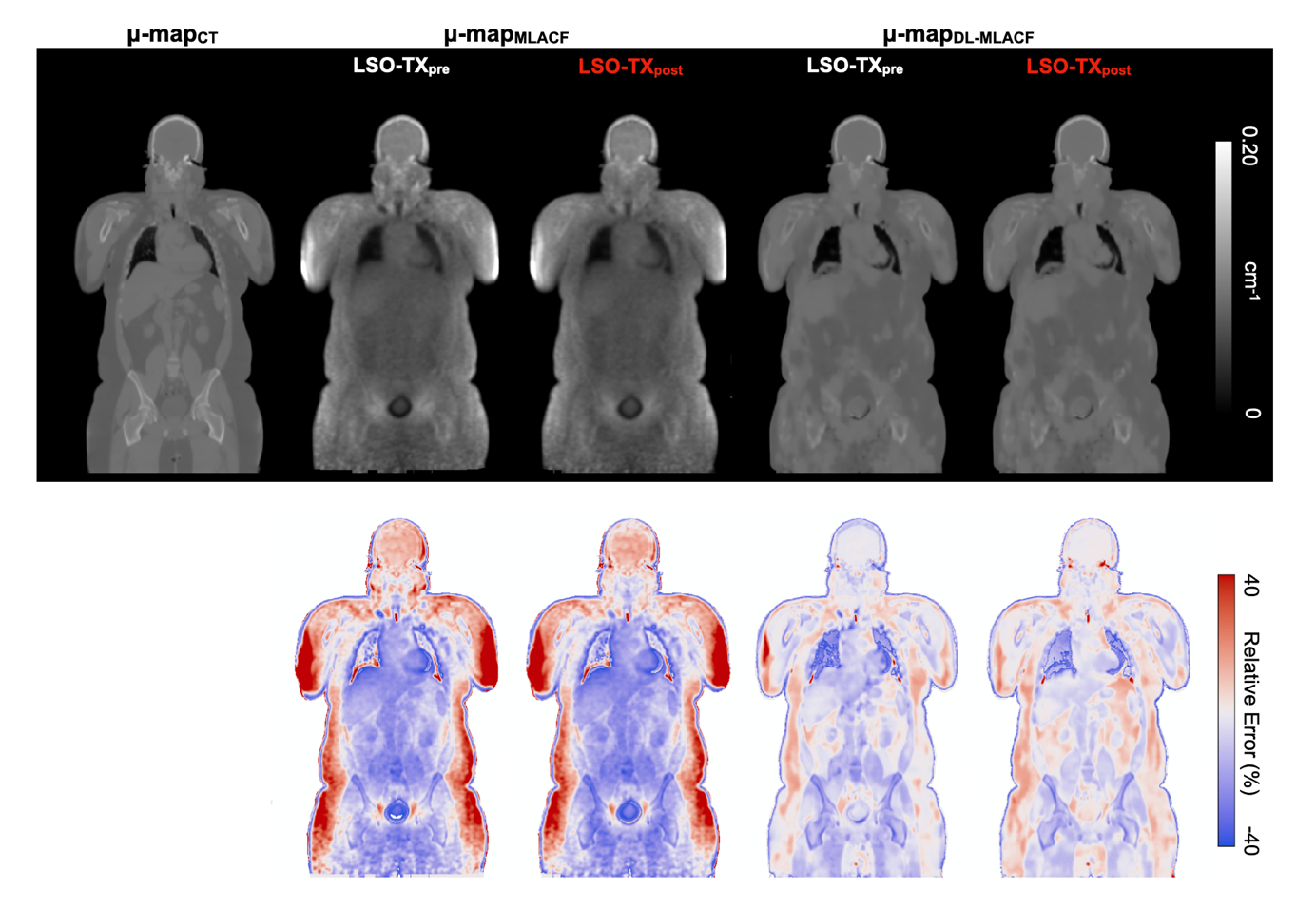
**

**Supplementary figure 1:** Top row: Attenuation maps of a subject with a BMI of 42 kg/m^2^ generated using the CT-, MLACF- and deep learning enhanced MLACF-based methods. Attenuation maps from pre- and post-injection LSO-TX acquisitions are shown separately. Bottom row: voxelwise maps of relative error distribution of MLACF- and DL-MLACF-based µ-maps relative to CT-based µ-map.

**
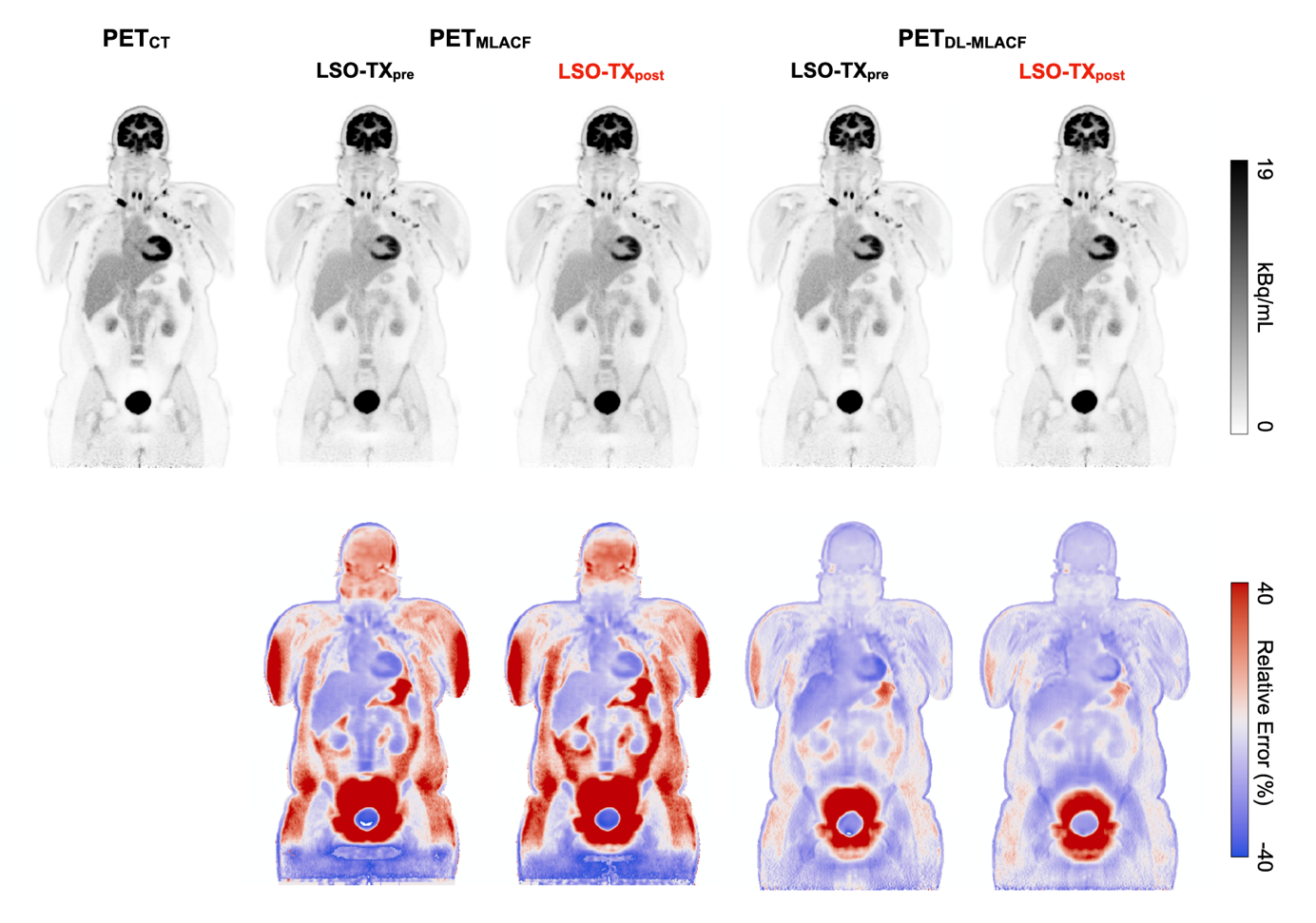
**

**Supplementary figure 2:** Top row: PET images of a subject BMI of 42 kg/m^2^ reconstructed using the CT-, MLACF- and deep learning enhanced MLACF- based attenuation maps. PET images reconstructed using MLACF- and DL-based µ-maps generated using pre- and post-injection LSO-TX data are shown separately. Bottom row: voxelwise maps of relative error distribution of PET images relative to the PET image reconstructed using the CT-based µ-map.

**A**

**B**

**C**

**D**

**Supplementary figure 3:** Scatter plots of whole-body rMAE% against patient BMI values for (A) PET_MLACF-PRE_, (B) PET_MLACF-POST_, (C) PET_DL-MLACF-PRE_, and (D) PET_DL-MLACF-POST_. Each plot includes a regression line. Spearman correlation r_s_ and p-values are also reported in each subplot.


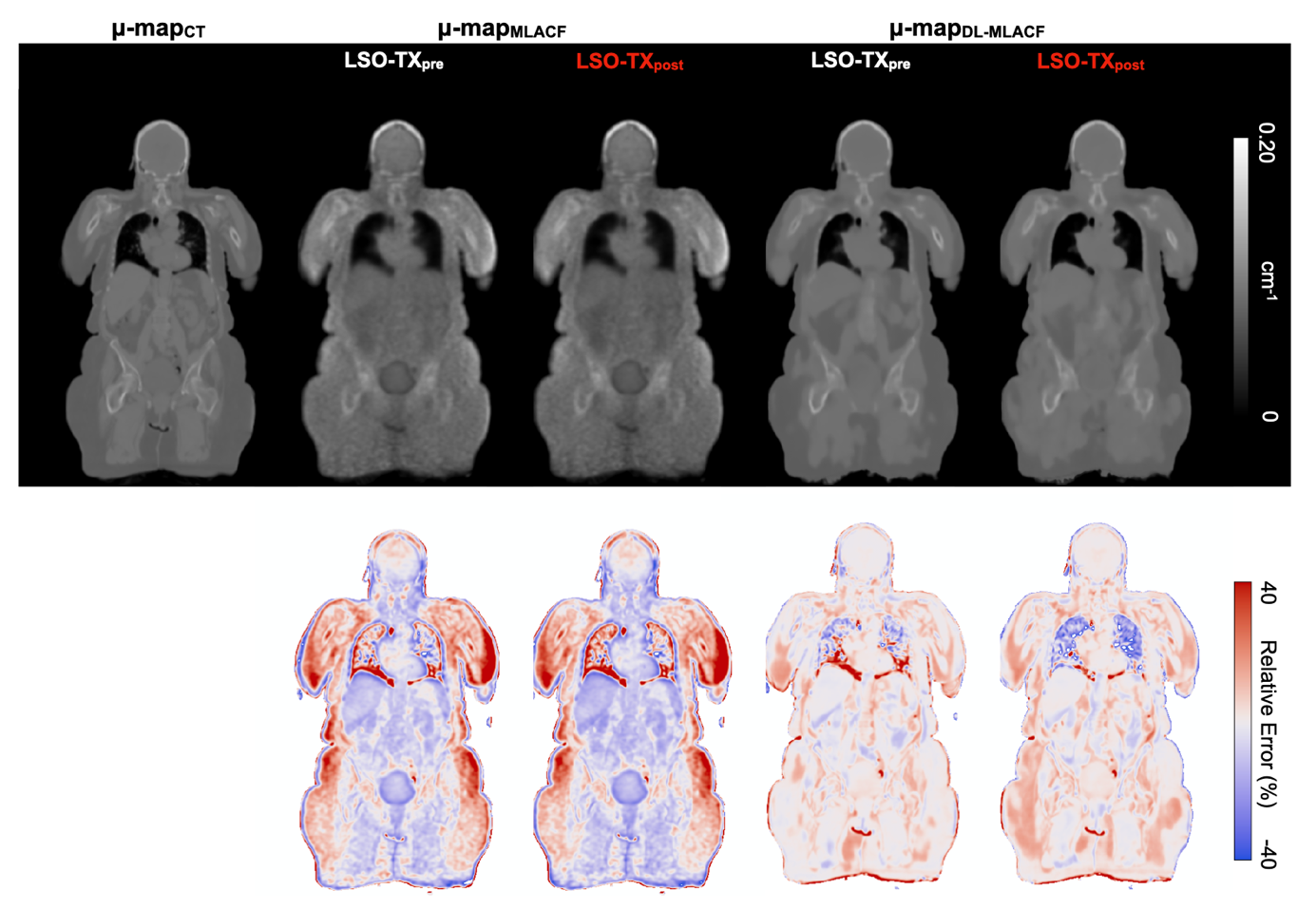


**Supplementary figure 4:** Top row: Attenuation maps of a subject with a BMI of 32.5 kg/m^2^ generated using the CT-, MLACF- and deep learning enhanced MLACF-based methods. Attenuation maps from pre- and post-injection LSO-TX acquisitions are shown separately. Bottom row: voxelwise maps of relative error distribution of MLACF- and DL-MLACF-based µ-maps relative to CT-based µ-map.


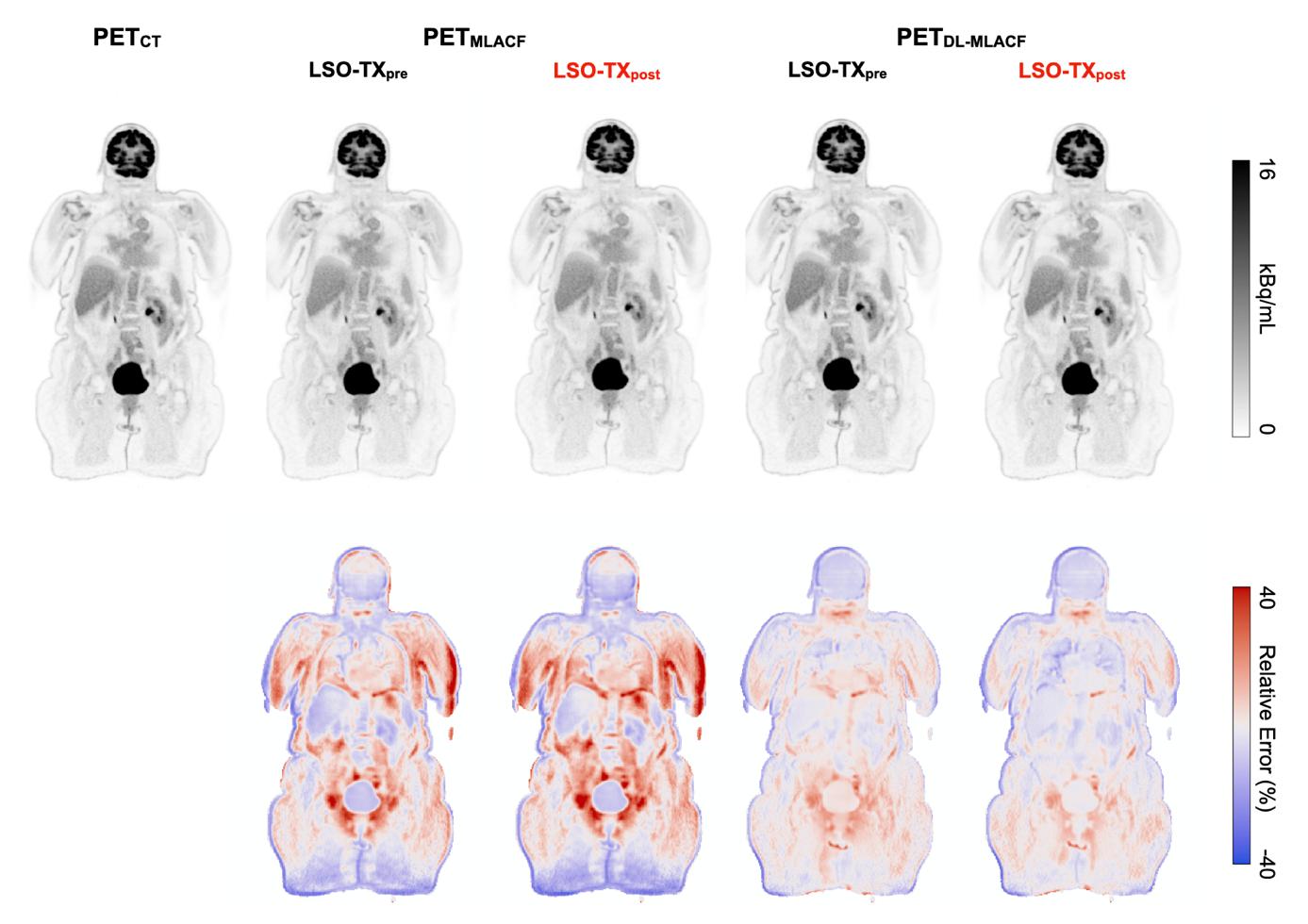


**Supplementary figure 5:** Top row: PET images of a subject BMI of 32.5 kg/m^2^ reconstructed using the CT-, MLACF- and deep learning enhanced MLACF- based attenuation maps. PET images reconstructed using MLACF- and DL-based µ-maps generated using pre- and post-injection LSO-TX data are shown separately. Bottom row: voxelwise maps of relative error distribution of PET images relative to the PET image reconstructed using the CT-based µ-map.
